# Macro–Micro Properties and Damage Model of Calcareous Sand Stabilized by Sulfoaluminate and Ferroaluminate Cements Under Different Water Environments

**DOI:** 10.3390/ma19091793

**Published:** 2026-04-28

**Authors:** Minghao Gu, Liang Cao, Peng Cao, Zhifei Tan, Ziyu Wang, Jingwei Ma

**Affiliations:** 1 College of Architecture and Civil Engineering, Beijing University of Technology, Beijing 100124, China; gurockyadou@163.com (M.G.); liangcao988@126.com (L.C.);; 2 Yazhou Bay Innovation Institute, Hainan Tropical Ocean University, Sanya 572025, China; zywang@hntou.edu.cn; 3 College of Marine Science and Technology, Hainan Tropical Ocean University, Sanya 572022, China; 4 Safety Assessment Guarantee Department, Beijing 100073, China

**Keywords:** calcareous sand, sulfoaluminate cement, ferroaluminate cement, seawater, damage modeling, hydration products

## Abstract

Island reef road construction faces a complex marine service environment characterized by high salinity and high humidity. Meanwhile, rapid construction and prompt subgrade repair are urgently required, creating a strong demand for novel calcareous-sand-based stabilization materials that combine excellent mechanical performance with resistance to seawater erosion. To this end, this study developed an early-strength cemented calcareous-sand reinforcement material for road base construction. Sulfoaluminate cement (SAC) and ferrite-aluminate cement (FAC), both featuring rapid setting/early strength development and superior corrosion resistance, were used to cement calcareous sand (CS) and to investigate its mechanical and microstructural characteristics under different water environments. Unconfined compressive strength tests (UCS) showed that SC-CS and FC-CS could meet subgrade requirements at 1 d and 7 d, with SC-CS and FC-CS reaching 3.12 MPa and 3.44 MPa at 1 d, and 3.26 MPa and 3.67 MPa at 7 d, respectively, under seawater SS conditions. Seawater mixing and immersion were found to promote the early strength and stiffness development of both SC-CS and FC-CS, with a more pronounced effect observed for FC-CS. Based on experimental results, a damage model for the stabilized specimens was established with a fitting accuracy of R^2^ > 0.97. This constitutive model accurately describes the stress–strain relationship of the material and quantitatively characterizes its damage evolution. Microscopic XRD and SEM analyses indicated that the main hydration product in freshwater-cured specimens was ettringite, and the interparticle connection of CS was dominated by bridging through rod-like ettringite. In contrast, under seawater conditions, the ettringite content decreased, while hydrotalcite and calcium aluminate hydrate increased, forming massive and lamellar bridging products. Compared with SC-CS, the bridging structure in FC-CS was denser. Moreover, the compactness of the bridging structure not only affected its mechanical properties but also governed the movement mode of CS particles, thereby influencing the damage evolution and failure mode of the specimens. The findings provide theoretical support for the construction needs of island road.

## 1. Introduction

Island highways are critical elements of transportation networks, supporting offshore resource development and emergency response [1]. Because of logistical constraints and compressed schedules, calcareous sand (CS)—abundant on many islands—is often used as subgrade fill [2]. Yet CS is formed by the accumulation of coral debris and shell debris; it has high porosity and low strength [3], so its direct use can lead to serious engineering failures. In addition, island regions are subjected to long-term erosion from complex marine environments and often confronted with emergent engineering tasks (e.g., post-storm emergency repairs [4]). These scenarios require subgrade materials that develop adequate strength within short periods, enabling rapid construction and early opening. Therefore, methods to stabilize CS and provide high early strength are urgently needed.

Current approaches to stabilize CS include microbially induced calcium carbonate precipitation (MICP) [5], enzyme-induced carbonate precipitation (EICP) [6], polyurethane foam adhesive (PFA) [7], and ordinary Portland cement (OPC) stabilization [8]. MICP and EICP strengthen CS by promoting calcium carbonate precipitation [9]. However, their effectiveness can be limited. In MICP, microbial survival under extreme temperature, salinity, or pH is poor, and the precipitates are often non-uniformly distributed [10,11]. In EICP, the enzyme solutions can exhibit limited permeability, dispersibility, and stability [12,13]. PFA bonds CS grains primarily through encapsulation [14], resulting in low overall strength of reinforced CS [15]. However, it infiltrates internal CS pores poorly, yielding low overall posttreatment strength [15]. Moreover, its high cost and limited environmental durability—especially in marine conditions—constrain practical use [16]. Compared with the three reinforcement methods discussed above, OPC-based reinforcement has been widely used in island and reef road engineering. OPC improves the mechanical performance of cementitious matrices through the filling and bridging actions of hydration products, primarily calcium silicate hydrate (C-S-H) and portlandite [17]. However, in seawater, Mg^2+^ and SO_4_^2−^ attack converts C-S-H and portlandite into non-cementitious magnesium silicate hydrates and expansive CaSO_4_ [18,19]. As a result, the mechanical properties of OPC-reinforced CS deteriorate markedly [20]. Moreover, the high CO_2_ emissions associated with OPC manufacture pose a pressing challenge for environmentally responsible island infrastructure [21,22]. Due to the shortage of freshwater resources in island and reef regions, the use of seawater for mixing subgrade materials has become an inevitable trend. However, abundant ions in seawater (such as Mg^2+^, SO_4_^2−^, Cl^−^) will further erode the cementitious system and degrade the mechanical properties and durability of stabilized calcareous sand specimens [23]. In summary, to adapt to the rapid construction and long-term service of island reef road engineering, it is urgent to find cementitious materials with excellent early strength and corrosion resistance for calcareous sand (CS) reinforcement.

Aluminate cement, which offers high early strength and resistance to seawater corrosion, is promising alternative to OPC for reinforcement applications. By mineralogical composition, aluminate cements are classified as sulfoaluminate cement (SAC) and ferroaluminate cement (FAC) [24,25]. SAC is dominated by ye’elimite (C_4_A_3_$\bar{S}$), belite (C_2_S), and anhydrite (C$\bar{S}$) [26,27]. FAC additionally contains higher fractions of ferrite phases (C_4_AF, C_6_AF_2_) [28]. Unlike ordinary Portland cement (OPC), the main hydration products of SAC and FAC are ettringite (C_6_A$\bar{S}$_3_H_32_) and monosulfoaluminate (C_4_A$\bar{S}$H_12_) [29,30]. These hydrates provide high early strength in both binders. Besides their high early-strength characteristics, existing studies have demonstrated that SC and FC also exhibit favorable corrosion resistance in seawater environments. Guo et al. [31] found that the monosulfoaluminate phase in SC plays a more critical role in reducing the chloride diffusion coefficient than ettringite and C_3_S. Cheng et al. [32] reported that Fe(OH)_3_ and AH_3_ gels formed during FC hydration can effectively fill internal pores and improve matrix compactness, making its resistance to gas permeability and chloride ion erosion superior to that of OPC. In seawater, SO_4_^2−^ and Ca^2+^ promote the formation and stabilization of ettringite [33,34], enhancing resistance to seawater attack. Furthermore, relative to OPC, SAC reduces CO_2_ emissions during clinkering by approximately 25–35% [35,36]. Moreover, compared with SAC, FAC requires a lower clinkering temperature, leading to further reductions in CO_2_ emissions [37]. Accordingly, using SC and FC to stabilize CS is promising for delivering high early-age strength and good durability in subgrade applications while reducing atmospheric impacts. However, to our knowledge, no study has reinforced CS with SAC or FAC and simultaneously evaluated macro- and microstructural properties under seawater mixing and immersion. Furthermore, the mechanisms by which SAC and FAC strengthen CS across different aqueous environments remain insufficiently understood, hindering optimization of mix designs for varied service conditions. In practice, reinforcement systems must satisfy pavement structural design requirements [38] and enable prediction of subgrade settlement [39]. To this end, damage models for reinforced specimens in different water environments need to be developed to provide theoretical support for engineering practice.

To fill these gaps, this study aims to reveal the macro- and microstructural evolution and strengthening mechanisms of calcareous sand (CS) modified by sulphoaluminate cement (SAC) and ferroaluminate cement (FAC) in different aqueous environments, and establish corresponding damage models to provide theoretical guidance for engineering practice.This study investigates the macro- and microstructures of SC/FC-CS across different aqueous environments, develops a damage model for the reinforced material, and elucidates the reinforcement mechanisms under varying water conditions. First, mechanical properties were evaluated using unconfined compressive strength (UCS) tests. Subsequently, a damage model was formulated by combining a hyperbolic constitutive model with a smooth damage-evolution function to obtain damage parameters. X-ray diffraction (XRD), scanning electron microscopy (SEM), and energy-dispersive X-ray spectroscopy (EDS) were then used to characterize hydration products, microstructure, and elemental compositions of SC/FC-CS. Finally, correlations among mechanical parameters, damage parameters, and hydration-product contents were analyzed, and reinforcement mechanisms of SAC and FAC under different aqueous environments were interpreted. The findings provide practical guidance for accelerating island transportation infrastructure construction and improving emergency response.

## 2. Methodology

### 2.1. Experiments

#### 2.1.1. Raw Materials

**Figure 1:**
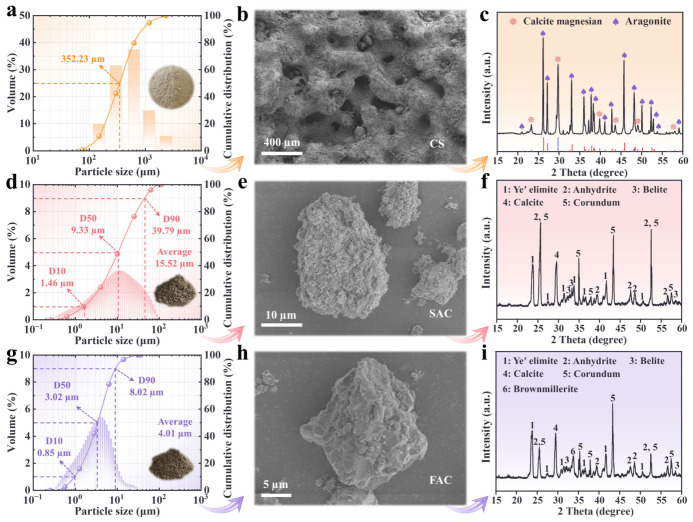
Physical and chemical properties of raw materials. (**a**) Grading curve of CS. (**b**) Morphological characterization of CS particle. (**c**) Mineral components of CS. (**d**) Grading curve of SAC. (**e**) Morphological characterization of SAC particle. (**f**) Mineral components of SAC. (**g**) Grading curve of FAC. (**h**) Morphological characterization of FAC particle. (**i**) Mineral components of FAC.

The physicochemical properties of CS, SAC, and FAC are summarized in Figure 1. CS was sourced from a reef area in the South China Sea; its particle-size distribution is shown in Figure 1a. Figure 1b shows the surface morphology of CS particles. The particle surfaces exhibit abundant pores and distinct coralline skeletal textures. The CS is dominated by aragonite (65.08%) and magnesian calcite (34.92%). SAC and FAC were commercial products (Zhucheng Jiuji Building Materials Co., Ltd., Zhucheng, Shandong, China). The particle-size distribution and microstructure of SAC are shown in Figure 1d and Figure 1e, respectively. SAC has an average particle size of 15.52 µm and exhibits aggregate-like mineral textures. Major phases in SAC were quantified by the Rietveld method with 20 wt% corundum (α-Al_2_O_3_) added as a standard [40] (Figure 1f). The cement primarily comprises ye’elimite (53.67%), anhydrite (23.11%), belite (15.96%), and calcite (4.86%). The particle-size distribution and microstructure of FAC are presented in Figure 1g,h. FAC has an average particle size of 4.01 µm and shows similar aggregate-like textures. Using the same Rietveld protocol (Figure 1i), FAC was found to consist primarily of ye’elimite (45.72%), anhydrite (15.86%), belite (12.24%), brownmillerite (8.78%), and calcite (5.19%). Water for mixing and curing included freshwater from the Sanya Water Treatment Plant and seawater collected from Dadonghai. Concentrations of major seawater ions are listed in Table 1. Citric acid (99 wt%), used as a set retarder, was purchased from Tianjin Zhiyuan Chemical Reagent Co., Ltd. (Tianjin, China).

#### 2.1.2. Specimen Preparation Process

Building on prior research [41], this study adopted a cement-to-calcareous sand ratio (C/CS) of 15% to achieve a favorable balance between mechanical performance and engineering economy, and a water-to-total mass ratio (W/M) of 12% to ensure adequate workability for construction and efficient cement hydration. The test specimens were cylindrical with a diameter of 39.1 mm and a height of 80 mm. The macroscopic and microscopic properties of CS reinforced with two types of cement were analyzed under different water conditions by varying the type of water used for mixing and curing. To prevent premature setting during specimen preparation, citric acid at 0.2% by mass of cement was incorporated into the mixing water [42]. The detailed material compositions for each experimental group are presented in Table 2. In this study, the freshwater mixing and immersion condition is denoted as FF, freshwater mixing with seawater immersion as FS, and seawater mixing and immersion as SS.

**Figure 2:**
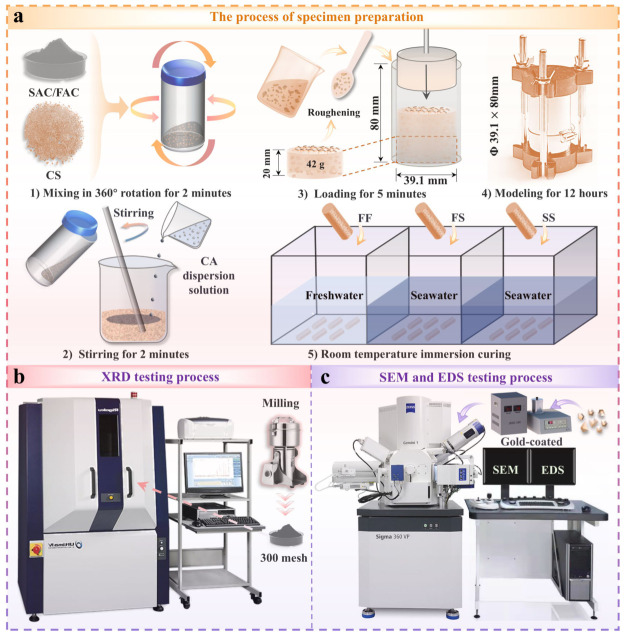
Specimen preparation and testing process. (**a**) Specimen preparation process. (**b**) XRD testing process. (**c**) SEM and EDS testing process.

The specimen preparation procedure consisted of five steps, as illustrated in Figure 2a. (1) A fixed mass of cement and CS was poured into a plastic container and dry-mixed by rotating the container 360° for 2 min. (2) The dry mixture and citric acid solution were transferred to a beaker and stirred thoroughly for 2 min. (3) The mixture was placed into the mold in four layers over 5 min. Each layer weighed 42 g and was 20 mm thick during placement. (4) The prepared specimen was sealed with plastic wrap and cured at room temperature (28 °C) for 12 h. (5) After demolding, the specimens were immersed in freshwater and seawater, respectively, for saturated curing at ambient temperature until the designated ages (1, 7, and 28 days). To maintain a constant ion concentration, the immersion water was replaced every three days.

#### 2.1.3. Specimen Testing Process

To obtain stress–strain curves for each specimen group under different water conditions, UCS were conducted. UCS were performed on a fully automatic unconfined compression tester (TKA-WXY-10F; Nanjing TKA Instrument Co., Ltd., Nanjing China) at a loading rate of 0.3 mm/min. To ensure reproducibility, five replicate specimens were tested for each condition, and the mean unconfined compressive strength (q_ucs_) was reported. XRD, SEM, and EDS were used to characterize hydration products, microstructures, and elemental composition for each group. For XRD, portions of the samples were ground with a mineral pulverizer and sieved to 45 μm. XRD patterns were collected on an Ultima IV diffractometer (Rigaku Corporation, Tokyo, Japan) (Figure 2b). Measurements used Cu/Kα radiation, a step size of 0.01°, a scanning speed of 4.0° min^−1^ in 2θ, and a range of 5–60° 2θ. Mineral phases were identified using the Inorganic Crystal Structure Database, and their relative contents were quantified via the Rietveld method [43]. Selected bulk specimens were sputter-coated with gold, then examined by SEM (Sigma 360; Carl Zeiss AG, ZEISS, Oberkochen, Germany) equipped with an EDS system (AZtec X-MaxN 80; Oxford, UK) (Figure 2c).

### 2.2. Mechanical Stiffness Characterization and Establishment of Damage Model

To quantitatively characterize and compare the mechanical stiffness of each group of specimens, this study adopts the secant modulus E_50_ at 50% of the peak stress as the evaluation index. Subsequently, to describe the nonlinear elastoplastic response of the material and the stiffness degradation law during loading, a damage model incorporating a smooth damage evolution equation is established, providing theoretical support for the prediction of material mechanical behavior and engineering numerical simulation.

#### 2.2.1. Mechanical Stiffness Characterization

To compare stiffness across groups, the secant modulus at 50% of peak stress, $E_{50}$, was calculated using Equation (1) [44].

(1) $$E_{50}=\frac{\sigma_{50}}{\varepsilon_{50}}$$

Here, $\sigma_{50}$ denotes 50% of the peak q_ucs_, and $\varepsilon_{50}$ is the corresponding strain on the ascending branch of the stress–strain curve.

#### 2.2.2. Construction of the Damage Model

The hyperbolic model effectively captures the nonlinear stress–strain response of materials across both elastic and plastic regimes [45]. The model is expressed in Equation (2). At small strains, the stress–strain relation is approximately linear; as strain increases, the curve progressively approaches an asymptote. Owing to this progressive nonlinearity, the hyperbolic form is widely used in constitutive formulations for geotechnical materials [46,47].

(2) $$\sigma (\varepsilon )=\frac{E_{0}\varepsilon }{1+\beta \varepsilon }$$

Here, σ(ε) denotes stress, ε is strain, $E_{0}$ is the initial modulus, and β is a curvature parameter that governs the rate of transition from elastic to plastic behavior. Physically, β determines the nonlinear curvature of the stress–strain curve, a larger β corresponds to an earlier and faster transition from linear elasticity to plastic yielding.

However, real materials exhibit not only nonlinear elastic-plastic responses but also damage, which must be considered. Although the hyperbolic model effectively describes the stress–strain response of undamaged materials, it does not capture the stiffness degradation that accompanies damage evolution [48]. To address this limitation, the hyperbolic model must be augmented. Damage mechanics models describe stiffness degradation and the associated changes in mechanical properties as damage evolves [49]. Therefore, integrating a damage model into the hyperbolic framework provides a more comprehensive representation of material behavior under load and yields a more accurate stress–strain description. Damage models commonly introduce a damage variable D(ε) to characterize the progression of material damage. D(ε)  typically increases with strain, thereby reducing stiffness in the stress–strain response [50]. Building on the hyperbolic model and D(ε), this study formulated the following constitutive model for the reinforced material:

(3) $$\sigma (\varepsilon )=\left(\begin{array}{c} 1-D(\varepsilon ) \end{array}\right)\frac{E_{0}\varepsilon }{1+\beta \varepsilon }$$

In traditional damage models, *D*(ε) is commonly defined using exponential or power laws [51,52]:

(4) $$D(\varepsilon )=1-\exp\left[-{\left(\frac{\varepsilon -\varepsilon_{0}}{\varepsilon_{d}}\right)}^{m}\right]$$

Here, ε_0_ represents the initial damage threshold strain corresponding to the onset of stiffness degradation. ε_d_ controls the overall degree of stiffness reduction, with larger values leading to more severe stiffness loss. m is the damage evolution exponent that determines the rate of damage accumulation and stiffness degradation with increasing strain.

However, conventional evolution laws often introduce discontinuities or slope breaks in the stress–strain curve at the initiation strain $\varepsilon_{0}$ [53]. These irregularities can destabilize computations, especially numerical simulations [54]. To mitigate this, this study embeds a smooth ramp function, ramp(x,s), into D(ε), ensuring continuity and differentiability near $\varepsilon_{0}$. The ramp function ramp(x,s) is defined as:

(5) $$\mathrm{ramp}(x,s)=0.5\left(x+\sqrt{x^{2}+s^{2}}\right)$$

Here, $x\;=(\varepsilon -\varepsilon_{0})$ denotes the strain increment driving damage evolution. s is a smoothing parameter that enhances numerical stability and enforces C^1^ continuity [55], controlling the smoothness of the strain field near the initiation point. It is set to 5% of the strain at peak stress, providing a smoother transition into the damage regime as the strain approaches its maximum. The final form of D(ε) is given by:

(6) $$D(\varepsilon )=1-\exp\left[-{\left(\frac{\mathrm{ramp}(\varepsilon -\varepsilon_{0},s)}{\varepsilon_{d}}\right)}^{m}\right]$$

## 3. Results

This chapter presents test results of SC-CS and FC-CS under different water environments via UCS, damage model fitting, XRD and SEM-EDS measurements. It analyzes the macroscopic mechanical properties, damage evolution, hydration product compositions and microstructural characteristics of the specimens, and reveals the regulatory effect of seawater on their performance and structure.

### 3.1. UCS Test Results

**Figure 3:**
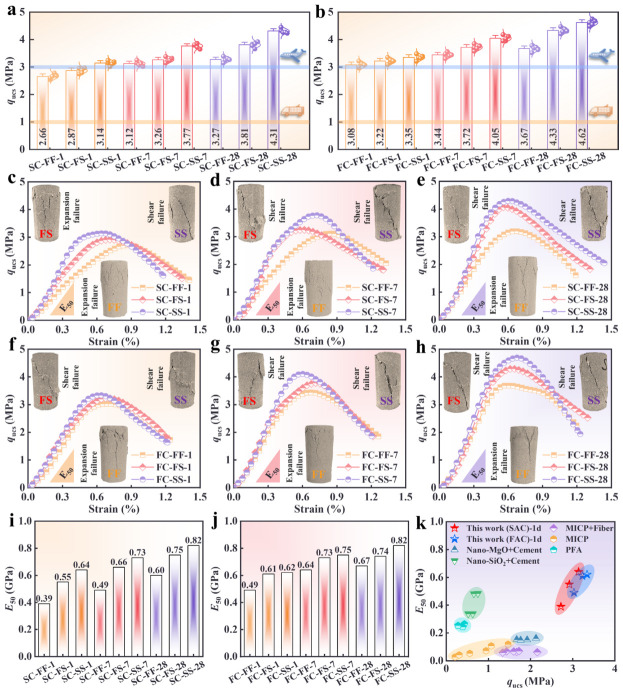
UCS test results. (**a**) q_ucs_ of SC-CS. (**b**) q_ucs_ of FC-CS. (**c**–**e**) Failure modes and stress–strain curves of SC-CS: (**c**) Soaking time 1 day; (**d**) Soaking time 7 days; (**e**) Soaking time 28 days. (**f**–**h**) Failure modes and stress–strain curves of FC-CS: (**f**) Soaking time 1 day; (**g**) Soaking time 7 days; (**h**) Soaking time 28 days. (**i**) E_50_ of SC-CS. (**j**) E_50_ of FC-CS. (**k**) Comparison of different reinforcement methods.

The UCS results for all specimen groups are presented in Figure 3. Figure 3a,b present q_ucs_ for SC-CS and FC-CS, respectively, under different water conditions and immersion durations. The q_ucs_ increased monotonically as immersion time increased, with all groups exhibiting low standard deviations (ranging from 0.08 to 0.1 MPa), indicating high test repeatability. The small standard deviations observed in the UCS results indicate low data variability and excellent experimental reproducibility, confirming the reliability of the test data. Seawater mixing and subsequent immersion increased the q_ucs_ for both SC-CS and FC-CS. In contrast, Wang et al. [20] reported that seawater mixing and immersion reduced the later-age strength of CS reinforced with OPC. These comparisons indicate that SAC and FAC are more suitable than OPC for reinforcing CS in seawater environments. Across all conditions, FC-CS achieved slightly higher q_ucs_ than SC-CS did. This difference is attributed to C_4_AF in FAC, which promotes rapid AFt formation [56] and yields additional iron-rich gel products [32]. Additionally, the yellow and blue lines in Figure 3a,b denote the 7-day q_ucs_ requirements for cement-stabilized base materials specified in Chinese highway [57] and civil airport runway [58] standards, respectively. For SC-CS, specimens in all three water conditions met the highway-base requirement after 1 day of curing, and those in the SS condition also satisfied the airport-runway requirement. For FC-CS, specimens in all three water conditions met both the highway-base and airport-runway requirements after 1 day. Thus, using SAC and FAC to reinforce CS can expedite road-construction schedules.

The failure patterns and the corresponding stress–strain curves are shown in Figure 3c–h. Under FF conditions, all groups exhibited compression-expansion (bulging) failure, particularly in SC-CS after 1 and 7 days of immersion. After seawater immersion, the failure mode shifted to oblique shear failure. Expansion failure is attributed to a relatively loose internal structure, which facilitates particle slip and rearrangement under compression [59]. In contrast, shear failure arises when interparticle displacements are constrained; under compression, this constraint promotes the formation of inclined shear cracks [60]. These observations indirectly indicate that seawater immersion enhances internal compaction. For SC-CS (Figure 3c–e), seawater immersion resulted in higher initial (tangent) stiffness than freshwater immersion, with the effect being most pronounced when seawater was used for mixing (SS) rather than immersion alone (FS). A similar trend was observed for FC-CS (Figure 3f–h), although the effect was slightly less pronounced due to the higher early strength of FAC.

Figure 3i,j present *E*_50_ for SC-CS and FC-CS, respectively. Seawater immersion significantly increased stiffness in both systems. Moreover, the stiffness gain of SC-CS was more pronounced than that of FC-CS. This suggests that seawater ions may promote the formation of stiffness-enhancing reaction products. In FC-CS, these products were generated in greater quantities. Additionally, the 1-day strength and stiffness of SC-CS and FC-CS were compared with those of other reinforcement methods (Figure 3k). The comparison included nano-MgO + cement (28 d) [61], nano- SiO_2_ + cement (28d) [17], MICP + fiber [62], MICP [63], and PFA [64]. At 1 day, SC-CS and FC-CS outperformed the foregoing methods in both strength and stiffness. Compared with OPC-reinforced CS in [20], the 7 d UCS of SC-CS and FC-CS under SS condition increased by 45.2% and 58.9%, with E50 rising by 38.7% and 42.3%, respectively, proving that SAC/FAC can effectively resist seawater ion erosion and avoid stiffness degradation of OPC-based materials. As shown in Figure 3k, the 1 d E50 of FC-CS under SS condition reaches 0.82 GPa, which is 2.1 times that of MICP + fiber [62], 2.3 times that of PFA [64] and 1.2 times that of nano-SiO_2_ modified OPC [17] at 28 d. This remarkable early strength and stiffness advantage makes SAC/FAC-reinforced CS more suitable for emergency repair and rapid construction of island reef roads. In addition, unlike the continuous expansion failure of OPC-reinforced CS in seawater [20], SC-CS/FC-CS transforms from expansion failure to shear failure under seawater immersion, indicating seawater improves their internal compactness, a unique mechanical response unreported in existing studies. These findings support the application of SC-CS and FC-CS in road-base construction to deliver dual benefits: accelerated schedules and improved strength.

### 3.2. Model Fitting Results and Damage Parameter Analysis

**Figure 4:**
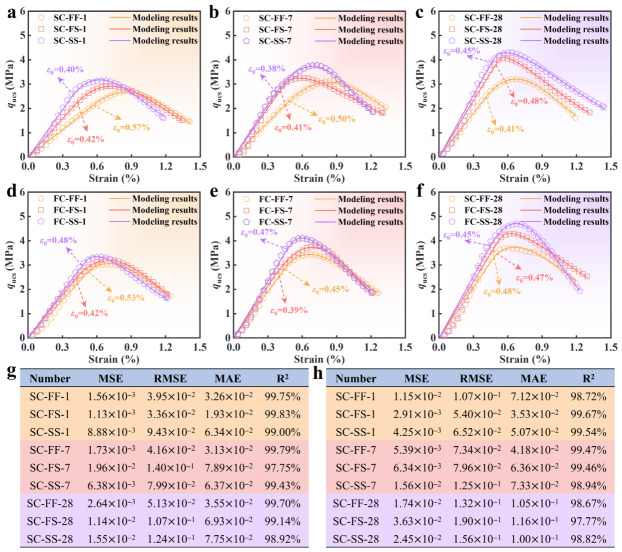
Comparison of damage model fitting results with experimental results. (**a**–**c**). Comparative results of SC-CS at different immersion times: (**a**) 1 d; (**b**) 7 d; (**c**) 28 d. (**d**–**f**). Comparative results of FC-CS at different immersion times: (**d**) 1 d; (**e**) 7 d; (**f**) 28 d. (**g**) Performance indices of damage model fitting to SC-CS. (**h**) Performance indices of damage model fitting to FC-CS.

The damage model developed was fitted to the stress–strain curves presented in Figure 4. The fitted curves reproduced the stress–strain responses of both SC-CS and FC-CS specimens. The agreement was particularly strong for the damage-initiation strain ($\varepsilon_{0}$) and the subsequent stiffness degradation. Furthermore, all fits achieved $R^{2}$ > 0.97 and had low values for mean-squared error (MSE), root-mean-squared error (RMSE), and mean absolute error (MAE) [65]. Collectively, these metrics confirmed that the model reliably predicted the stress–strain behavior of both specimen types. Overall performance was strong; however, the fit deteriorated at large strains for some specimens. For specimens exhibiting complex damage evolution, incorporating additional material parameters or adopting a more advanced damage formulation may improve high-strain fits.

**Figure 5:**
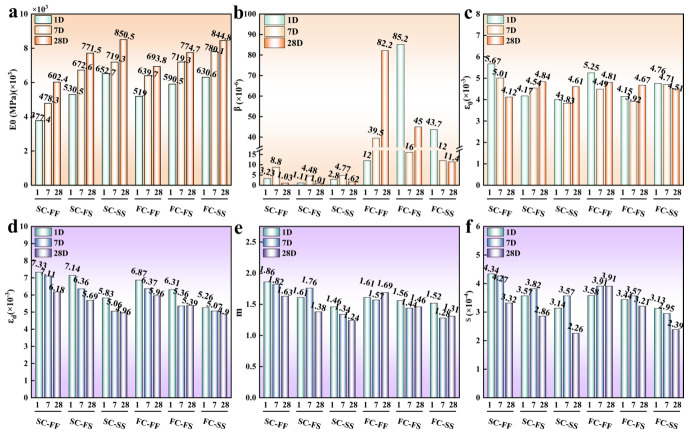
Damage model parameters of different specimens. (**a**) $E_{0}$ results of SC-CS and FC-CS specimens under different immersion times. (**b**) β results of SC-CS and FC-CS specimens under different immersion times. (**c**) $\varepsilon_{0}$ results of SC-CS and FC-CS specimens under different immersion times. (**d**) $\varepsilon_{d}$ results of SC-CS and FC-CS specimens under different immersion times. (**e**) m results of SC-CS and FC-CS specimens under different immersion times. (**f**) s results of SC-CS and FC-CS specimens under different immersion times.

Figure 5 summarizes the damage parameters for each fitted curve. Both seawater mixing and immersion increased E_0_ for SC-CS and FC-CS across immersion times. This trend is consistent with the E_50_ variation reported in Section 4.1, supporting the model’s validity. The parameter β shows little dependence on immersion time or water environment. This insensitivity likely reflects complex damage evolution within the material. Nevertheless, β is consistently higher in FC-CS than in SC-CS, suggesting faster stiffness evolution under increasing stress. Similarly, the damage-onset strain $\varepsilon_{0}$ shows no clear trend across specimens. The scatter likely arises from nonuniform reinforcement distribution, which introduces local defects and randomizes damage initiation. After seawater mixing and immersion, E_0_ increased, whereas the damage-expansion strain $\varepsilon_{d}$, damage index m, and damage sensitivity decreased. These changes indicate an enhanced load-bearing capacity that delays entry into the plastic zone during early elastic deformation. Consequently, with further straining, both the rate and sensitivity of damage accumulation diminished. Moreover, damage accumulation and propagation became more governed by bulk stiffness than by localized stress concentrations. Overall, seawater mixing and immersion stiffened SC-CS and FC-CS and suppressed damage accumulation and propagation. The parameter variations in the damage model partially capture how aquatic exposure influences compressive damage in reinforced materials. These findings provide theoretical guidance for assessing damage in SC-CS and FC-CS in engineering practice.

### 3.3. XRD Test Results

**Figure 6:**
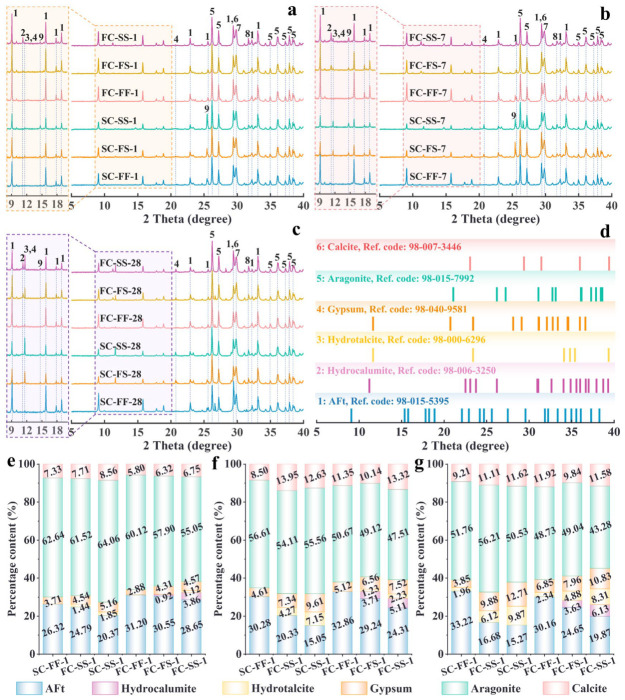
XRD test results. (**a**–**c**) Mineral components of each specimen group: (**a**) 1 day; (**b**) 7 days; (**c**) 28 days. (**d**) Reference card codes for major minerals. (**e**–**g**) Major mineral content of each group: (**e**) 1 day; (**f**) 7 days; (**g**) 28 days. (1: AFt; 2: Hydrocalumite; 3: Hydrotalcite; 4: Gypsum; 5: Aragonite; 6: Calite; 7: Calcite magnesium; 8: Halite; 9: Anhydrite.).

Figure 6 presents the mineral fraction characteristics of SC-CS and FC-CS under different immersion times and aqueous environments. After 1 day of immersion (Figure 6a), the main detectable hydration product in both SC-CS and FC-CS specimens across the three aqueous environments was ettringite (3CaO·Al_2_O_3_·3CaSO_4_·32H_2_O, AFt). No distinct diffraction peaks were observed for Al(OH)_3_, Fe(OH)_3_, or C-(A)-S-H gels, due to their amorphous nature. The formation processes of these products are shown in Equations ((7)–(10)). Furthermore, the diffraction peak intensities of AFt in FC-CS were consistently higher than those in SC-CS under the same aqueous environment. This is primarily due to the faster reaction rate of ferrite phases in FC, which accelerates the formation of Aft [56]. This explains why the early intensity of FC-CS is higher than that of SC-CS. Notably, the diffraction peak intensities of AFt in both FS and SS environments were lower than those in the FF environment, particularly in SC-CS specimens. This is primarily because Mg^2+^ in seawater reacts with OH^−^ in the specimens, forming Mg(OH)_2_ gel. This process weakens the specimen’s alkaline environment, thereby inhibiting AFt formation.

(7) $$C_{4}A_{3}\;\bar{S}+2C\;\bar{S}H_{2}+34H\to C_{6}A{\;\bar{S}}_{3}H_{32}+2AH_{3}$$

(8) $$C_{4}A_{3}\;\bar{S}+18H\to C_{4}A\;\bar{S}H_{12}+2AH_{3}$$

(9) $$2C_{2}S+4H\to C_{3}S_{2}H_{3}+\mathrm{CH}$$

(10) $$3C_{4}\mathrm{AF}+3C\;\bar{S}H_{2}+30H\to C_{6}A{\;\bar{S}}_{3}H_{32}+\mathrm{CH}+FH_{3}$$

After 7 days of immersion (Figure 6b), the AFt diffraction peak intensity under FF conditions increased further. By contrast, in SC-CS exposed to FS and SS, the AFt peak intensity decreased, whereas the hydrotalcite peaks increased. These changes likely reflect AFt decomposition and its reaction with Mg(OH)_2_ gel to form hydrotalcite as specimen alkalinity decreases. In contrast, in FC-CS, the AFt peak intensity remained nearly unchanged, while the hydrocalumite peak increased. This pattern indicates that Fe(OH)_3_ gel formed in FC-CS exerts a protective effect, stabilizing AFt. This gel likely hinders the ingress of external Mg^2+^, thereby favoring hydrocalumite over hydrotalcite. However, after 28 days (Figure 6c), the AFt peak intensity in FC-CS decreased in both FS and SS, whereas the hydrotalcite peak increased markedly. This suggests that, with prolonged seawater exposure, Mg^2+^ gradually penetrates the Fe(OH)_3_ protective layer and weakens specimen alkalinity. Consequently, AFt decomposes, promoting hydrotalcite formation. Additionally, no diffraction peaks attributable to Ca(OH)_2_ were detected in any specimen group. This finding is consistent with previous studies [32,66]. It likely reflects further reactions of Ca(OH)_2_ with Al(OH)_3_ and Fe(OH)_3_ gels to form AFt and Fe-AFt, as shown in Equations ((11)–(13)). Moreover, Fe-AFt shows greater stability, elasticity, and sulfate-attack resistance than Aft [67]. These attributes likely contribute to the higher strength of FC-CS relative to SC-CS under identical conditions. In addition, halite diffraction peaks were detected in all specimen groups exposed to FS and SS. This observation indicates that seawater ions fully penetrated the specimens, thereby altering the assemblage of hydration products.

(11) $$C_{6}AF_{2}+\mathrm{CH}+C\;\bar{S}H_{2}+H\to C_{6}\left(A,F\right){\;\bar{S}}_{3}H_{32}+FH_{3}$$

(12) $$3\mathrm{CH}+3C\;\bar{S}H_{2}+AH_{3}+20H\to C_{6}A{\;\bar{S}}_{3}H_{32}$$

(13) $$3\mathrm{CH}+3C\;\bar{S}H_{2}+AH_{3}+2FH_{3}+17H\to C_{6}\left(A,F\right){\;\bar{S}}_{3}H_{32}$$

To further quantify the impact of different generator contents on specimen mechanical properties, the relative mineral contents in each group were determined using the Rietveld method. Figure 6d shows the card numbers for the major minerals in the Inorganic Crystal Structure Database. Figure 6e–g present the relative mineral contents in both specimens under varying aqueous environments and immersion times. As immersion time increased, the relative AFt content in SC-CS under FF conditions gradually rose to 33.22%. The AFt content in FC-CS under FF conditions reached 32.86% at 7 days but slightly decreased at 28 days. In both FS and SS environments, AFt content in both specimens gradually decreased with increasing immersion time. However, the contents of hydrocalumite, hydrotalcite, and gypsum gradually increased. This further suggests that hydrocalumite, hydrotalcite, and gypsum contribute to the enhancement of specimen mechanical properties at later stages.

### 3.4. SEM-EDS Test Results

**Figure 7:**
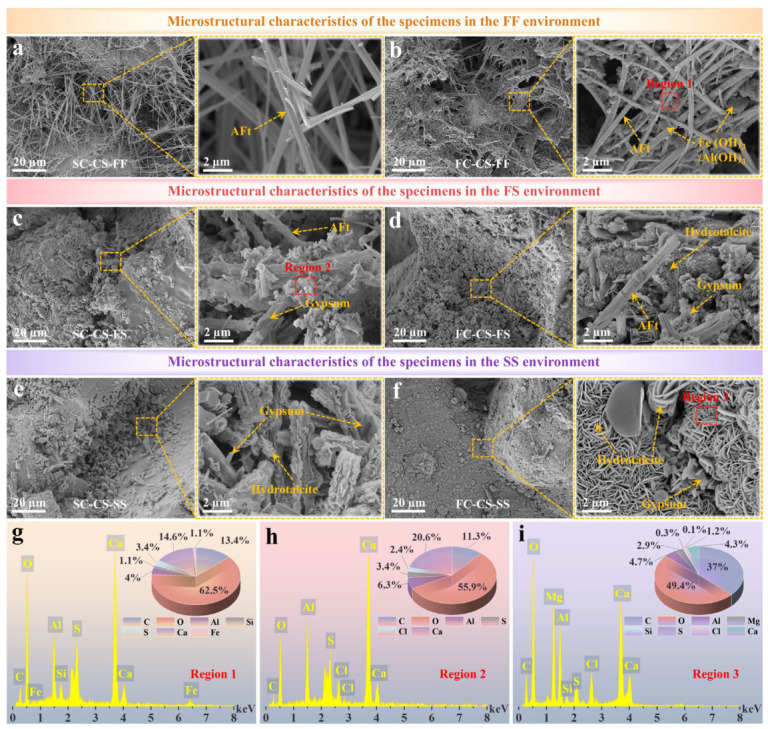
Microstructural characteristics and elemental distributions of each specimen group under various aqueous environments. (**a**) Microstructure of SC-CS under the FF environment. (**b**) Microstructure of FC-CS under the FF environment. (**c**) Microstructure of SC-CS under the FS environment. (**d**) Microstructure of FC-CS under the FS environment. (**e**) Microstructure of SC-CS under the SS environment. (**f**) Microstructure of FC-CS under the SS environment. (**g**–**i**) Elemental distribution maps of selected regions: (**g**) Region 1; (**h**) Region 2; (**i**) Region 3.

Figure 7 illustrates the microstructural characteristics and elemental distribution in specific regions of each specimen group at 28 days. Under the FF environment, the CS particles in SC-CS (Figure 7a) and FC-CS (Figure 7b) specimens were bridged by numerous rod-like hydration products. However, while only rod-like products were observed between CS particles in SC-CS, FC-CS exhibited rod-like products interspersed with a greater abundance of bulk products, resulting in a denser bridging structure. In addition, a limited number of rod-like products were present on the surface of CS particles in SC-CS, whereas FC-CS displayed a more extensive coverage. Densification between CS particles was significantly enhanced in the FS environment for both SC-CS (Figure 7c) and FC-CS (Figure 7d). In this condition, the number of rod-like products between CS particles decreased significantly, and the interstitial spaces were predominantly filled by massive products. Furthermore, compared to the FF environment, both specimens in the FS environment exhibited more extensive hydration product coatings on CS particle surfaces, leading to nearly complete encapsulation. In the SS environment (Figure 7e,f), the bridging structures between CS particles were denser than those in the FF and FS environments. Notably, numerous lamellar structures were present within the bridging zones, forming near-continuous connections between adjacent particles. This effect was especially evident in FC-CS, where both the CS particle surfaces and interparticle gaps were fully covered by lamellar products. Collectively, the accumulation of hydration products on particle surfaces and the development of dense bridging structures provide a microstructural basis for the macroscopic behavior of the specimens.

Additionally, Figure 7g–i show the elemental contents of three regions in the SEM maps above. The presence of Al and S in region 1 (Figure 7g), combined with XRD results and morphological features, confirmed that the rod-like structures correspond to AFt. In FC-CS, these rod-like formations also contained Fe, consistent with previous reports of Fe-bearing Aft [68]. Elemental analysis of region 2 (Figure 7h) indicated that the bulk products surrounding AFt primarily consisted of sulfoaluminate phases. A small amount of Cl was also present in these minerals, suggesting the participation of Cl- from seawater in hydration product formation, thereby contributing to the denser bridging structure. In region 3 (Figure 7i), Mg was identified within the lamellar structures, confirming that these products are predominantly composed of hydrotalcite.

## 4. Discussion

To reveal the microstructure mechanism governing macroscopic mechanical properties, this study establishes correlations between mechanical parameters and the content of hydration products. By investigating the hydration mechanism, the relationship between the dense bridging structure among CS particles and the reinforcement performance as well as failure mode is elucidated.

### 4.1. Correlation Between q_ucs_, $\varepsilon_{d}$ and AFt Content

**Figure 8:**
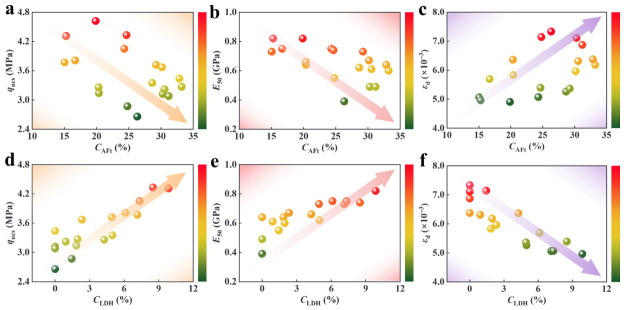
Relationship between the mechanical properties and product content of the specimens. (**a**) *q*_ucs_ and *C*_Aft_ (AFt relative content). (**b**) *E*_50_ and *C*_Aft_. (**c**) $\varepsilon_{d}$ and *C*_AFt_. (**d**) *q*_ucs_ and *C*_LDH_ (LDH content). (**e**) *E*_50_ and *C*_LDH_. (**f**) $\varepsilon_{d}$ and *C*_LDH_.

To further investigate the relationship between macroscopic properties and microscopic components, correlations were established between mechanical parameters (q_ucs_, E_50_, $\varepsilon_{d}$) and the contents of key hydration products (C_AFt_ and C_LDH_), as illustrated in Figure 8. Here, C_AFt_ represents the relative content of AFt in the specimen, while C_LDH_ denotes the content of Layered Double Hydroxides (LDH), including hydrocalumite and hydrotalcite. As shown in Figure 8a–c, q_ucs_ and E_50_ exhibited a negative correlation with C_AFt_, whereas $\varepsilon_{d}$ showed a positive correlation. These results indicate that a high AFt content accelerates specimen damage, leading to premature failure at the ultimate load, which can be attributed to the dual detrimental effects of AFt on the microstructural integrity of the cementitious matrix. This behavior is primarily attributed to the rod-like structure of AFt. Under external loading, the high-aspect-ratio AFt rods act as intrinsic stress concentrators, particularly at their ends, promoting the accumulation of internal micro-damage. AFt undergoes substantial volume expansion (exceeding 120% relative to reactants). Excessive AFt precipitation in confined pores generates high crystallization pressure, inducing microcracking at the CS particle–matrix interface and weakening the bonding phase. This causes the specimen to reach a critical state before the theoretical peak load is attained. In contrast, Figure 8d–f show that q_ucs_ and E_50_ were positively correlated with C_LDH_, while $\varepsilon_{d}$ was negatively correlated. This suggests that a higher LDH content inhibits premature damage accumulation and significantly enhances the ultimate load-bearing capacity of the specimens. The improved mechanical performance can be ascribed to the unique lamellar structure of LDH phases. Compared to the rod-like structure of AFt, LDH provides a larger interfacial contact area, facilitating more uniform stress distribution throughout the specimen and thereby contributing to enhanced overall strength and stiffness.

### 4.2. Mechanisms of Strength Enhancement and Damage Evolution

**Figure 9:**
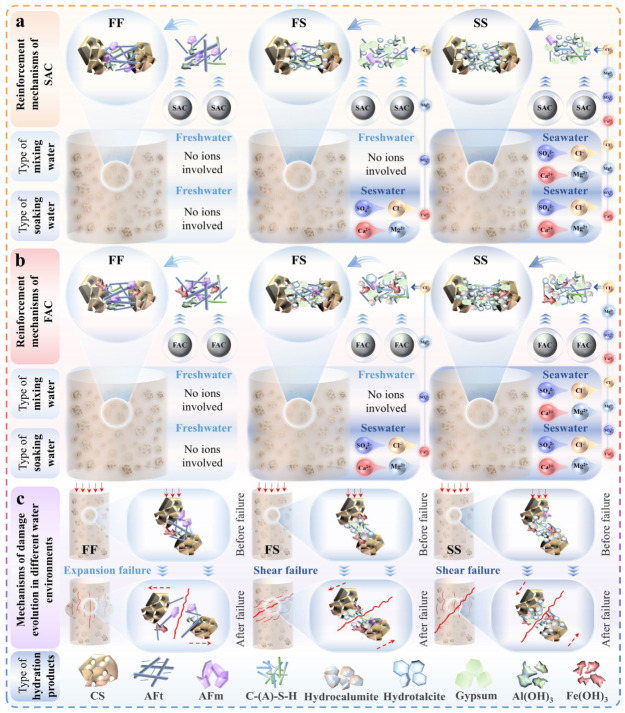
Mechanisms of strength enhancement and damage evolution. (**a**) Strength enhancement mechanisms of SC-CS in three water environments. (**b**) Strength enhancement mechanisms of FC-CS in three water environments. (**c**) Micro-damage mechanisms of specimens under three water environments.

Figure 9 illustrates the reinforcement and micro-damage mechanisms of the specimens under three aqueous environments (FF, FS, SS). As shown in Figure 9a, AFt, AFm, Al(OH)_3_, and C-(A)-S-H gels were rapidly generated during SAC hydration in the FF environment, filling the interstitial spaces between CS particles. In this system, rod-like AFt and fibrous C-(A)-S-H gels primarily formed bridging networks between particles, while Al(OH)_3_ gels and sheet-like AFm filled the voids among these products. This synergistic interaction effectively bonded the CS particles, enabling efficient load transfer under external loading and accounting for the early strength and stiffness observed in SC-CS. In the FS environment, ions such as Cl^−^, SO_4_^2−^, Mg^2+^, and Ca^2+^ from seawater penetrated the specimen, promoting the formation of hydrotalcite and gypsum. This led to a denser bridging structure between CS particles compared to the FF condition. Further enhancement was observed in the SS environment, where the bridging structures became even more compact, explaining the higher 28-day strength of specimens cured under the SS environment. Figure 9b illustrates the reinforcement mechanism of FAC on CS in the three aqueous environments. While similar to SAC in enhancing interparticle bonding, FAC exhibited a faster hydration rate, resulting in denser bridging structures at equivalent curing ages. The formation of hydrocalumite, hydrotalcite, Fe-containing AFt, and Fe(OH)_3_ gels further contributed to improved the toughness and strength of the bridging structure. This explains why FC-CS demonstrates superior mechanical performance relative to SC-CS. Notably, ionic supplementation from seawater in both FS and SS environments promoted greater accumulation of hydration products on CS particle surfaces, strengthening the interface between particles and the binding phases.

## 5. Conclusions

This study develops a novel calcareous-sand reinforcement material using SAC and FAC as cementitious materials, tailored to complex marine environments and engineered for rapid construction and seawater erosion resistance. It systematically investigates how mixing and soaking water types affect the mechanical properties of SC-CS and FC-CS, reveals the reinforcement and damage evolution mechanisms of sulfoaluminate and ferrite-aluminate cements on calcareous sand under three aqueous conditions based on mineral composition and microstructural characteristics, and establishes a theoretical model to describe the damage evolution of the specimens. The main conclusions are as follows:

(1) Both SC-CS and FC-CS exhibit excellent early-age mechanical properties under seawater mixing and seawater soaking conditions, confirming the feasibility and engineering advantages of using SC and FC to stabilize calcareous sand for island roadbed engineering in seawater environments. Seawater mixing and soaking can improve the q_ucs_ and $E_{50}$ of SC-CS and FC-CS at 1 day, enabling them to meet the application requirements of highway base courses and airport runway base courses.
(2) The proposed hyperbolic damage constitutive model can accurately reproduce the stress–strain behavior of SC-CS and FC-CS under compressive loading, with a coefficient of R^2^ exceeding 0.98. The model can capture the damage initiation strain $\varepsilon_{0}$ and stiffness degradation characteristics of the materials. With the increase in specimen stiffness, both the damage propagation strain $\varepsilon_{d}$ and the damage exponent m decrease.
(3) The enhancement of mechanical properties in the seawater environment is attributed to the alteration of hydration product composition and interparticle bridging morphology in the cementitious system, thereby improving the compactness of the microstructure. The experimental results show that Mg^2+^ in seawater inhibits the formation of AFt. Therefore, specimens in the FF environment have the highest AFt content, and the interparticle bridging is dominated by rod-like AFt, resulting in low structural compactness. In contrast, the contents of hydrocalumite and hydrotalcite increase in FS and SS environments. Interparticle bridging in FS is dominated by flaky products, while that in SS is dominated by blocky products, leading to high material compactness. The compactness of the cemented structure between calcareous sand particles directly controls the particle movement pattern, thereby affecting the failure mode and damage evolution path of specimens.
(4) By establishing linear relationships between mechanical parameters (q_ucs_, $\varepsilon_{d}$, material stiffness E_50_) and the contents of micro-products (AFt and C_LDH_), this study clarifies the influence of hydration product content on macroscopic properties. Specifically, q_ucs_ and E_50_ are negatively correlated with AFt, while $\varepsilon_{d}$ is positively correlated with AFt. Conversely, q_ucs_ and E_50_ are positively correlated with C_LDH_, while $\varepsilon_{d}$ is negatively correlated with C_LDH_.

Despite revealing the macro–micro properties and damage evolution law of calcareous sand stabilized by sulfoaluminate/ferroaluminate cement, this study has certain limitations. Only short-to-medium term static immersion tests of 1, 7 and 28 days were conducted, and long-term durability tests under the coupled complex marine environments such as dry-wet cycles and dynamic traffic loads for the actual service conditions of island reef subgrades have not been carried out.

This study provides a reliable material scheme and theoretical support for roadbed, highway base and airport runway construction in island and reef engineering, and is of great practical significance for the efficient and durable construction of marine infrastructure.

## Figures and Tables

**Table 1:** Concentration of different ions in seawater.

Ion	${\mathrm{Cl}}^{-}$	${\mathrm{Na}}^{+}$	${\mathrm{SO}}_{4}^{2-}$	${\mathrm{Mg}}^{2+}$	${\mathrm{Ca}}^{2+}$	$K^{+}$	${\mathrm{HCO}}_{3}^{-}$	${\mathrm{Ba}}^{2+}$
Concentration/(mg·L^−1^)	18,537.27	11,237.31	2812.27	1289.23	432.27	452.86	157.39	0.28

**Table 2:** Type and proportion of materials in each group of specimens.

Number	C/CS	W/M	CA/C	Cement Type	Mixing Mode	Immersion Mode
SC-FF	15%	12%	0.2%	SAC	Freshwater	Freshwater
SC-FS	15%	12%	0.2%	SAC	Freshwater	Seawater
SC-SS	15%	12%	0.2%	SAC	Seawater	Seawater
FC-FF	15%	12%	0.2%	FAC	Freshwater	Freshwater
FC-FS	15%	12%	0.2%	FAC	Freshwater	Seawater
FC-SS	15%	12%	0.2%	FAC	Seawater	Seawater

## Data Availability

The original contributions presented in this study are included in the article. Further inquiries can be directed to the corresponding authors.

## Abbreviations

AFm	Monosulfoaluminate
AFt	Ettringite
CAFt	Relative content of ettringite
CLDH	Content of Layered Double Hydroxides
C/CS	Cement-to-calcareous sand ratio
CS	Calcareous sand
EDS	Energy-dispersive X-ray spectroscopy
EICP	Enzyme-induced carbonate precipitation
OPC	Ordinary Portland cement
PFA	Polyurethane foam adhesive
qucs	Unconfined compressive strength
RMSE	Root mean squared error
SAC	Sulfoaluminate cement
SC-CS	Calcareous sand stabilized by sulfoaluminate cement
E50	Secant modulus at 50% of the peak stress
FAC	Ferroaluminate cement
FC-CS	Calcareous sand stabilized by ferroaluminate cement
FF	Freshwater mixing and freshwater immersion
FS	Freshwater mixing and seawater immersion
MAE	Mean absolute error
MICP	Microbially induced calcium carbonate precipitation
MSE	Mean squared error
SS	Seawater mixing and seawater immersion
UCS	Unconfined compressive strength
W/M	Water-to-total mass ratio
XRD	X-ray diffraction
SEM	Scanning electron microscopy
